# Chitinase-3 like-protein-1, matrix metalloproteinase -9 and positive intracranial arterial remodelling

**DOI:** 10.3389/fnagi.2023.1154116

**Published:** 2023-04-06

**Authors:** Ming Tang, Dongyang Zhou, Junhui He, Hongying Bai, Qianqian Li, Hui Xu

**Affiliations:** ^1^Department of Neurology, The Second Affiliated Hospital of Zhengzhou University, Zhengzhou, China; ^2^Department of Cardiology, The Second Affiliated Hospital of Zhengzhou University, Zhengzhou, China

**Keywords:** cerebral small vessel disease, YKL-40, MMP-9, chitinase-3 like-protein-1, arterial remodelling

## Abstract

**Introduction:**

Positive intracranial arterial remodelling is a dilated lesion of the large intracranial vessels; however, its pathogenesis is currently unknown. Some studies have identified chitinase-3 like-protein-1 (YKL-40) and matrix metalloproteinase (MMP)-9 as circulating inflammatory factors involved in positive vascular remodelling. Herein, we aimed to investigate the relationship between changes in serum YKL-40 and MMP-9 levels and positive intracranial arterial remodelling in patients with cerebral small vessel disease (CSVD).

**Methods:**

A total of 110 patients with CSVD were selected. Patients with brain arterial remodelling (BAR) scores >1 times the standard deviation were defined as the positive intracranial artery remodelling group (*n* = 21 cases), and those with BAR scores ≤1 times the standard deviation were defined as the non-positive intracranial artery remodelling group (*n* = 89 cases). Serum YKL-40 and MMP-9 levels were measured using an enzyme-linked immunosorbent assay kit. Factors influencing positive intracranial artery remodelling using binary logistic regression analysis and predictive value of YKL-40 and MMP-9 for positive intracranial arterial remodelling in patients with CSVD were assessed by a subject receiver operating characteristic curve.

**Results:**

Statistically significant differences in serum YKL-40 and MMP-9 levels were observed between the positive and non-positive remodelling groups (*p* < 0.05). The integrated indicator (OR = 9.410, 95% CI: 3.156 ~ 28.054, P<0.01) of YKL-40 and MMP-9 levels were independent risk factors for positive intracranial arterial remodelling. The integrated indicator (OR = 3.763, 95% CI: 1.884 ~ 7.517, *p* < 0.01) of YKL-40 and MMP-9 were independent risk factors for positive arterial remodelling in posterior circulation, but were not significantly associated with positive arterial remodelling in anterior circulation (*p* > 0.05). The area under the curve for YKL-40 and MMP-9 diagnostic positive remodelling was 0.778 (95% CI: 0.692–0.865, *p* < 0.01) and 0.736 (95% CI: 0.636–0.837, p < 0.01), respectively.

**Discussion:**

Elevated serum YKL-40 and MMP-9 levels are independent risk factors for positive intracranial arterial remodelling in patients with CSVD and may predict the presence of positive intracranial arterial remodelling, providing new ideas for the mechanism of its occurrence and development and the direction of treatment.

## 1. Introduction

Positive intracranial arterial remodelling is a phenomenon in which large intracranial arteries dilate outwards for various reasons, causing an increase in the area of the canal wall (Jaminon et al., 2019). Although it is possible to maintain the lumen area to a certain extent and reduce the narrowing of the vessels, several studies have reported that positive remodelling increases plaque instability, that is, its risk of fragility, bleeding, and dislodgement, and is strongly associated with the development of ischaemic cerebrovascular events (Zhang et al., 2017; Yang et al., 2020). The mechanism by which positive intracranial arterial remodelling occurs has not been defined. Two theories are now generally accepted: firstly, long-term changes in haemodynamics, producing continuous positive vascular pressure, induce the production of large amounts of matrix metalloproteinases by vascular endothelial cells, causing wall remodelling (Cooke and Dzau, 1997). The second is the inflammatory response, plaque theory, where positive intracranial arterial remodelling is often accompanied by more atherosclerotic plaques (Kataoka et al., 2009). Inflammatory factor chitinase-3 like-protein-1(YKL-40) may contribute to intracranial atherosclerotic plaque formation by participating in cellular inflammatory chemotaxis, endothelial cell migration, and accelerated intracellular lipid accumulation (Park et al., 2012). Matrix metalloproteinase (MMP)-9 can lead to vascular remodelling by mediating vascular matrix fibrosis and promoting atherosclerosis (Pahk et al., 2019). Most previous studies on intracranial arterial remodelling are retrospective studies of stroke patients who were part of an endpoint event that had occurred and had diagnostic value but lacked ideas for preventive treatment (Sun J. et al., 2022; Kaur et al., 2023). The burden caused by prolonged dilatory remodelling of the vessel wall is transmitted from large vessels to small vessels. Positive intracranial arterial remodelling has been observed in patients with different imaging types of cerebral small vessel disease (CSVD) (Thijs et al., 2017; Squair et al., 2018; Chen et al., 2020). Therefore, this study provides a new perspective on the development of positive intracranial arterial remodelling by including patients with CSVD and multiple arteries in their anterior and posterior circulation and will provide new ideas for its prevention and treatment.

## 2. Materials and methods

### 2.1. Patients

**Figure 1:**
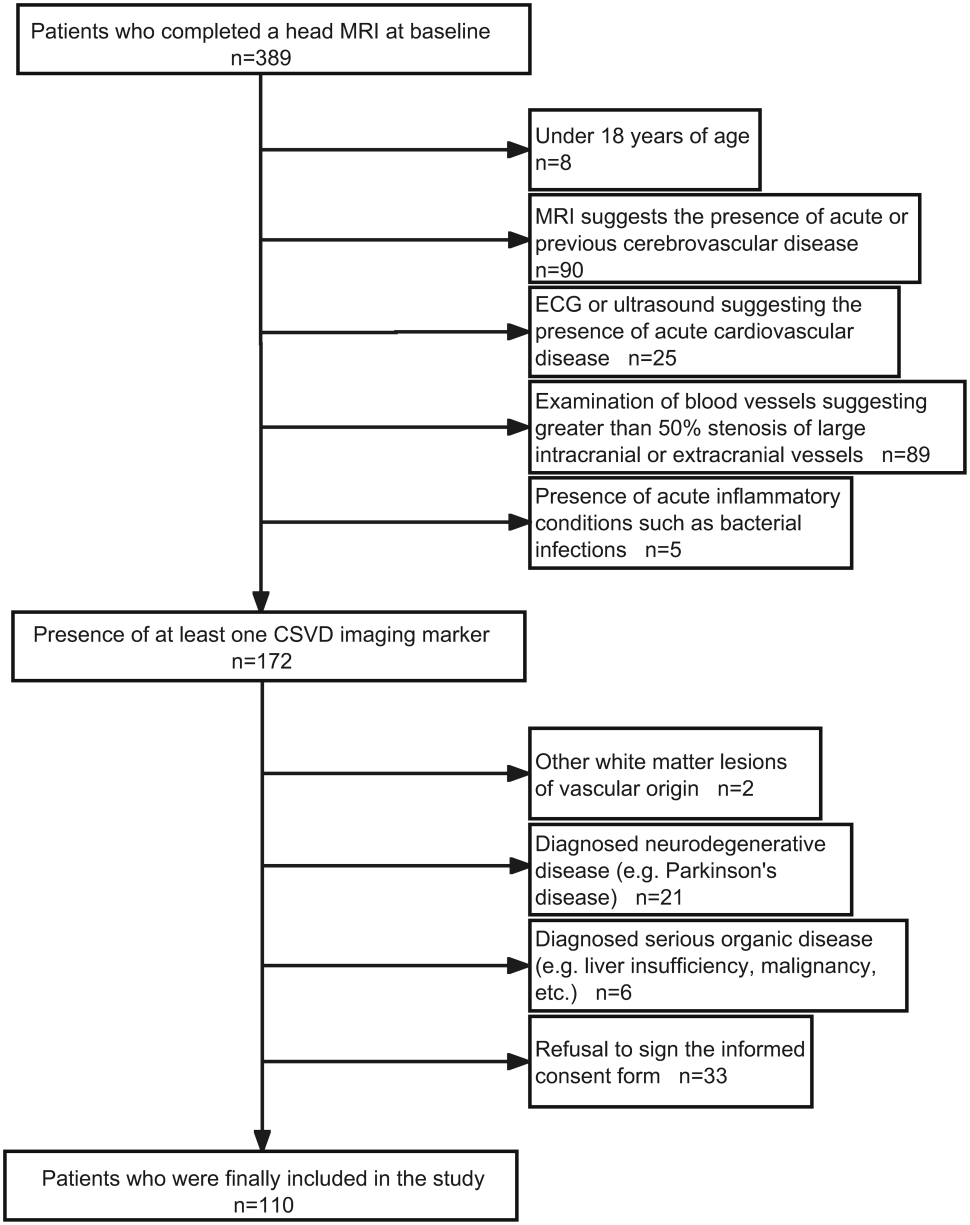
Admission flow chart.

110 patients with CSVD who attended the Second Affiliated Hospital of Zhengzhou University between December 2020 and October 2022 were included in this study, and all patients signed an informed consent form, we have created figure to explain the process of enrolling patients (Figure 1). This study was approved by the Ethics Committee of the Second Affiliated Hospital of Zhengzhou University (No. 2022194). The inclusion criteria were as follows: (1) meeting the diagnostic criteria of CSVD in the Chinese Diagnostic and Treatment Guidelines for Cerebral Small Vessel Disease 2020 (Chinese Society of and Chinese Stroke, 2022); (2) age > 18 years; (3) cooperation with perfect cranial magnetic resonance imaging (MRI) and serum collection; and (4) provision of informed consent for this study. The exclusion criteria were as follows: (1) magnetic resonance angiography (MRA) or cervical vascular ultrasound suggesting intracranial and extracranial large artery stenosis ≥50%; (2) patients with acute cardiovascular and cerebrovascular disease, such as cerebral infarction, cerebral haemorrhage, subarachnoid haemorrhage, and myocardial infarction; (3) patients with acute inflammatory response, such as fever and infection; (4) other non-vascular causes of cerebral white matter lesions; (5) previous history of cardiovascular and cerebrovascular disease; (6) severe organic diseases, hepatic and renal insufficiency, and malignant tumours; and (7) other neurological diseases, such as Parkinson’s disease and Alzheimer’s disease.

### 2.2. Data collection

Baseline patient information was collected, including gender, age, history of hypertension (previous definite diagnosis of hypertension or blood pressure ≥ 140/90 mmHg before MRI examination), history of diabetes mellitus (previous definite diagnosis of diabetes mellitus or fasting glucose ≥7 mmol/L before MRI examination), history of hyperlipidaemia (previous or definite diagnosis of hyperlipidaemia before MRI examination), history of smoking (previous or current smoking ≥1 cigarette per day), and history of alcohol consumption (alcohol intake ≥15 g/d). Collected laboratory data included total cholesterol (TC), triacylglycerol (TG), high-density lipoprotein cholesterol (HDL-C), low-density lipoprotein cholesterol (LDL-C), small and low-density lipoprotein cholesterol (sdLDL-C), and homocysteine (Hcy).

### 2.3. Detection of serological indexes

Fasting venous blood (5 mL) was collected in the morning within 48 h after the patients were admitted to the hospital. The serum was centrifuged and separated at room temperature for 2 h (1,000× *g*/15 min). The absorbance values of serum YKL-40 and MMP-9 at 450 nm were measured by enzyme-linked immunosorbent assay at the same room temperature, and the concentrations were calculated. The average concentration was used as the inclusion index.

### 2.4. Imaging

MRI examinations were completed within 1 week of admission using Siemens Skyra 3.0 T superconducting MRI equipment (Skyra3.0 T, Siemens, Munich, Germany). The main scanning sequences were time-of-flight magnetic resonance angiography (TOF MRA), T1-weighted imaging, T2-weighted imaging, fluid-attenuated inversion recovery, diffusion-weighted imaging, and susceptibility-weighted imaging.

**Figure 2:**
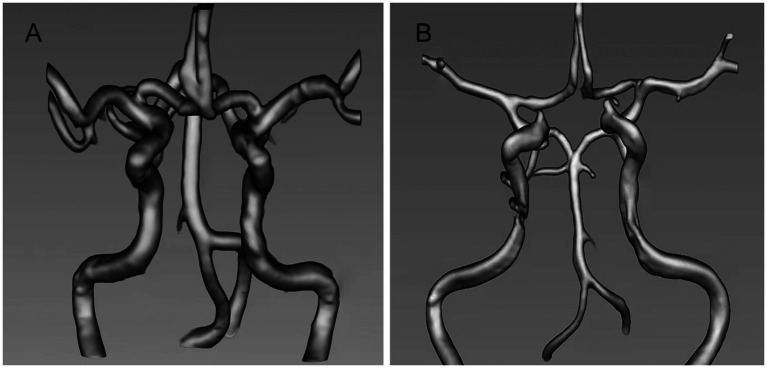
**(A)** Positive intracranial arterial remodelling. **(B)** Non-positive intracranial arterial remodelling.

Image analysis and grouping criteria images were processed by an experienced neurologist and an imaging physician. Raw data from TOF MRA sequences of all enrolled patients were imported into the open source medical image processing system three-dimensional (3D)-Slicer software (3D-Slicer 4.10.2, National Institutes of Health, USA) and 3D reconstruction and alignment of the large intracranial arteries was performed using Volume Rendering or Segment Editor modules. The maximum arterial diameters of the intracranial segment of the bilateral internal carotid arteries, the A1 segment of the bilateral anterior cerebral arteries, the M1 segment of the bilateral middle cerebral arteries, the P1 segment of the bilateral posterior cerebral arteries, the V4 segment of the bilateral vertebral arteries and the pontocerebral segment of the basilar arteries were measured separately. The diameter of arteries not visualized in the TOF MRA sequence was defined as 0. The patient’s overall intracranial arterial remodelling index was defined as the sum of the diameters of the 11 arteries measured divided by the total of 11. The anterior circulation remodelling index was defined as the sum of the diameters of the largest arteries measured in the intracranial segment of the bilateral internal carotid arteries, the A1 segment of the bilateral anterior cerebral arteries, and the M1 segment of the bilateral middle cerebral arteries divided by the total of 6. The posterior circulation remodelling index, defined as the brain arterial remodelling (BAR) score, is obtained by adding the maximum arterial diameters of the P1 segment of the posterior cerebral artery, the V4 segment of the vertebral artery, and the pontocerebral segment of the basilar artery and then dividing them by the total number of 5. The mean and standard deviation of the BAR were calculated and standardized to evaluate the remodelling of the intracranial arteries (Gutierrez et al., 2015, 2017, 2018). The mean and standard deviation of the BAR scores were calculated and standardized to assess intracranial arterial remodelling. Patients with BAR scores ≥1 times the standard deviation were defined as individuals with positive intracranial arterial remodelling; those with BAR scores <1 times the standard deviation were defined as individuals with non-positive intracranial arterial remodelling (Man and Xu, 2017; Figure 2). The patients were divided into two groups: positive and non-positive remodelling groups for all arteries, positive and non-positive remodelling groups for the anterior circulation, and positive and non-positive remodelling groups for the posterior circulation (Sun J. et al., 2022).

### 2.5. Statistical analysis

Statistical analysis was performed using SPSS version 26.0, and measurement data conforming to normal distribution were expressed as the mean ± standard deviation ( $\bar{x}$ ±s). Two independent samples t-test was used for comparison between groups. Measures that did not conform to a normal distribution were expressed as the median (interquartile range) [M(QR)], and the Mann–Whitney U test was used for comparison between groups. Statistical data were expressed as relative values (cases/%), and comparisons between groups were made using the χ^2^ test or Fisher’s exact test. Factor analysis was used to combine YKL-40, MMP-9 into one variable. Factors influencing positive intracranial arterial remodelling were analysed using binary logistic regression. The receiver operating characteristic curve was plotted, and the area under the curve was calculated to assess the diagnostic efficacy of YKL-40 and MMP-9 for positive intracranial arterial remodelling. Reproducibility analysis was used to calculate the intraclass correlation coefficients to assess the consistency of intracranial artery diameter measurements between the two physicians.

## 3. Results

### 3.1. Statistical description

A total of 110 patients who met the inclusion criteria were recruited with a mean age of 65 years, including 58 men and 52 women, 61 with hypertension, 21 with diabetes mellitus, 12 with hyperlipidaemia, 26 smokers, and 17 drinkers. Eleven arterial diameters were measured. The mean diameter of the overall intracranial artery was 2.9 ± 0.4 mm, the mean diameter of the anterior circulation artery was 3.2 ± 0.4 mm, and the mean diameter of the posterior circulation artery was 2.6 ± 0.5 mm. Twenty-one patients with positive overall arterial remodelling and 89 with non-positive remodelling were included. There were 19 patients with positive remodelling of the anterior circulation, 91 with non-positive remodelling of the anterior circulation, 20 with positive remodelling of the posterior circulation, and 90 with non-positive remodelling of the posterior circulation.

### 3.2. Analysis of positive and non-positive remodelling

#### 3.2.1. Analysis of differences between groups

The differences in gender, history of diabetes and alcohol consumption, TC, TG, HDL-C, LDL-C, sdLDL-C, and Hcy were not statistically significant (*p* > 0.05), whereas the differences in age, history of hypertension, history of hyperlipidemia, history of smoking, YKL-40, and MMP-9 were statistically significant (*p* < 0.05) between the positive and non-positive remodelling groups (Table 1).

**Table 1:** Comparison of positive and non-positive remodelling.

Projects	Non-positive remodelling (*N* = 89)	Positive remodelling (*N* = 21)	Statistical values	Value of *p*
Age [M(QR), year]	64(18.5)	72(10.5)	−2.039	0.041
Gender (M/%)	47(53)	11(52)	0.001^a^	0.972
Hypertension (cases/%)	45(50.6)	16(76.2)	4.518^a^	0.034
Diabetes mellitus (cases/%)	15(16.9)	6(28.6)	1.51^a^	0.219
Hyperlipidemia (cases/%)	6(6.7)	6(28.6)	8.331^a^	0.004
Smoking history (cases/%)	16(18.0)	10(47.6)	8.271^a^	0.004
Drinking history (cases/%)	11(12.4)	15(71.4)	3.418^a^	0.064
TC [( $\bar{x}$ ± *s*)，mmol/L]	4.13 ± 1.01	3.89 ± 0.83	1.052^b^	0.295
TG [M(QR), mmol/L]	1.09(0.84)	0.99(0.69)	−1.152	0.249
HDL-C [M(QR), mmol/L]	1.28(0.54)	1.27(0.55)	−0.43	0.667
LDL-C [M(QR), mmol/L]	2.38(1.34)	2.21(1.06)	−1.057	0.29
sdLDL-C [M(QR), mmol/L]	0.52(0.42)	0.53(0.30)	−0.791	0.429
Hcy [M(QR), mmol/L]	12.4(4.75)	11.5(6.5)	−0.719	0.472
YKL-40 [M(QR), ng/mL]	42.28(18.48)	55.22(16.74)	−3.959	<0.001
MMP-9 [M(QR), ug/mL]	161.34(59.17)	195.27(44.66)	−3.358	0.001

^a^is the *χ*^2^ value of the *χ*^2^ test. ^b^is the *t*-value of the two independent samples *t*-test, and the remaining statistic is the *z*-value of the Mann–Whitney U test.

#### 3.2.2. Risk factor analysis

A one-way binary logistic regression analysis (Model A) was performed with the statistically significant variables in Table 1 as independent variables and whether or not positive remodelling was the dependent variable, respectively. The results showed that age [odds ratio (OR) = 1.051, 95% confidence interval (CI): 1.001–1.103, *p* = 0.045], history of hypertension (OR = 0.32, 95% CI: 0.108–0.948, *p* = 0.040), hyperlipidemia (OR = 5.533, 95% CI: 1.573–19.471, *p* = 0.008), smoking (OR = 4.148, 95% CI: 1.506–11.422, *p* = 0.006), YKL-40 (OR = 1.079, 95% CI: 1.033–1.128, *p* = 0.001), MMP-9 (OR = 1.029, 95% CI: 1.012–1.046, *p* = 0.001), and integrated indicator (OR = 9.566, 95% CI: 3.665 ~ 24.966, *p* < 0.01) were all risk factors for positive remodelling. After correcting for age, history of hypertension, hyperlipidaemia, and smoking (Model B), the results showed that integrated indicator (OR = 9.410, 95% CI: 3.156 ~ 28.054, *p* < 0.01) were independent risk factors for positive remodelling (Table 2).

**Table 2:** Binary logistic regression analysis of risk factors for positive remodelling.

	Model A			Model B		
Projects	OR	95% CI	Value of *p*	OR	95% CI	Value of *p*
Age	1.051	1.001 ~ 1.013	0.045	–	–	–
Hypertension	0.32	0.108 ~ 0.948	0.04	–	–	–
Hyperlipidemia	5.533	1.573 ~ 19.471	0.008	–	–	–
Smoking history	4.148	1.506 ~ 11.442	0.006	–	–	–
YKL-40	1.079	1.033 ~ 1.128	0.001	1.078	1.016 ~ 1.143	0.013
MMP-9	1.029	1.012 ~ 1.046	0.001	1.029	1.006 ~ 1.052	0.013
Integrated indicators	9.566	3.665 ~ 24.966	<0.01	9.410	3.156 ~ 28.054	<0.01

Model A is a one-way logistic regression analysis, and Model B is after correction for age, history of hypertension, history of hyperlipidaemia, and history of smoking.

#### 3.2.3. Receiver operating characteristic curve analysis

**Figure 3:**
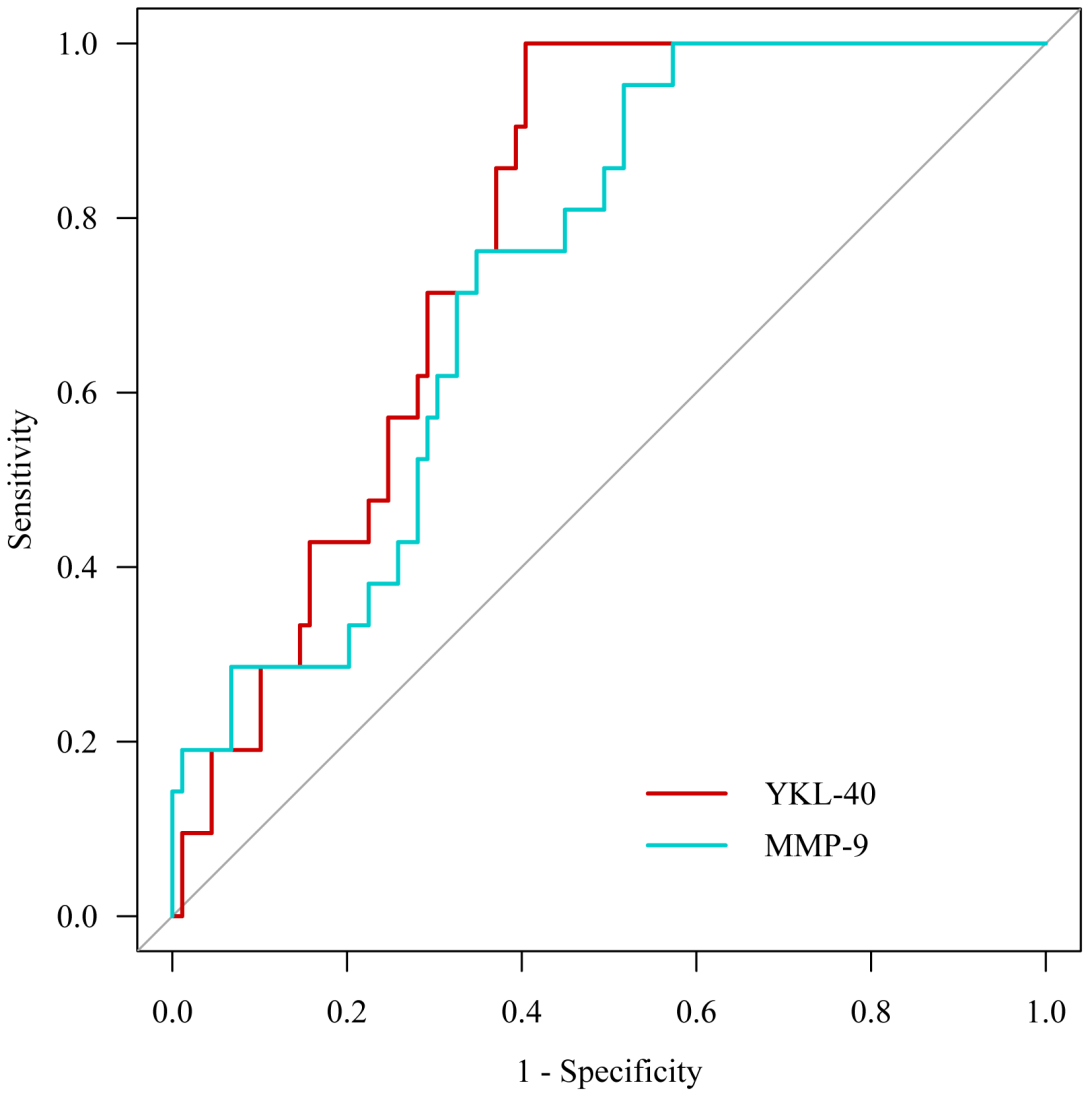
Receiver operating characteristic curve analysis.

After plotting the receiver operating characteristic curves, we observed that the areas under the YKL-40 and MMP-9 curves were 0.778 (95% CI: 0.692–0.865, *p* < 0.001) and 0.736 (95% CI: 0.636–0.837, *p* = 0.001), respectively (Figure 3).

### 3.3. Analysis of pre- and post-loop positivity and non-positivity remodelling

#### 3.3.1. Analysis of differences between groups

The differences in age, sex, history of hypertension, diabetes, hyperlipidaemia, alcohol consumption, TC, TG, HDL-C, LDL-C, sdLDL-C, and Hcy were not statistically significant (*p* > 0.05) between the anterior circulation positive and the non-positive remodelling groups. However, the differences in smoking history were statistically significant (*p* < 0.05) (Table 3). Also, the differences in age, sex, history of hypertension, hyperlipidaemia, alcohol consumption, TC, TG, HDL-C, LDL-C, sdLDL-C, and Hcy were not statistically significant between the remodelling groups (*p* > 0.05), whereas the differences in the history of diabetes, YKL-40, and MMP-9 were statistically significant (*p* < 0.05) (Table 4).

**Table 3:** Comparison of positive and non-positive remodelling in anterior circulation.

Projects	Non-positive remodelling (*N* = 91)	Positive remodelling (*N* = 19)	Statistical values	Value of *p*
Age [M(QR), year]	66(19)	68(16)	−0.265	0.791
Gender (M/%)	48(52.7)	10(52.6)	<0.001^a^	0.993
Hypertension (cases/%)	49(53.8)	12(63.2)	0.552^a^	0.458
Diabetes (cases/%)	20(22.0)	1(5.3)	–	0.116^c^
Hyperlipidemia (cases/%)	8(8.8)	4(21.1)	–	0.216^c^
Smoking history (cases/%)	18(19.8)	8(42.1)	4.340^a^	0.037
Drinking history (cases/%)	12(13.2)	5(26.3)	–	0.168^c^
TC [( $\bar{x}$ ± *s*), mmol/L]	4.10 ± 1.00	3.99 ± 0.90	0.46^b^	0.646
TG [M(QR), mmol/L]	1.07(0.74)	1.05(0.87)	−0.391	0.695
HDL-C [M(QR), mmol/L]	1.30(0.59)	1.20(0.39)	−0.518	0.604
LDL-C [M(QR), mmol/L]	2.34(1.12)	2.30(1.19)	−0.36	0.719
sdLDL-C [M(QR), mmol/L]	0.52(0.35)	0.53(0.50)	−0.123	0.902
Hcy [M(QR), mmol/L]	12.4(4.8)	12.2(6)	−0.558	0.577
YKL-40 [( $\bar{x}$ ± *s*), ng/mL]	45.73 ± 13.00	50.96 ± 12.41	−1.607^b^	0.111
MMP-9 [M(QR), ug/mL]	173.29(52.26)	187.84(47.02)	−1.301	0.193

^a^ is the *χ*^2^ value of the *χ*^2^ test, ^b^ is the *t*-value of the two independent samples *t*-test, ^c^ is the *p*-value of the Fisher exact test, and the remaining statistics are the *z*-values of the Mann–Whitney U test.

**Table 4:** Comparison of posterior circulation positive and non-positive remodeling in the posterior circulation.

Projects	Non-positive remodelling (*N* = 90)	Positive remodelling (*N* = 20)	Statistical values	Value of *p*
Age [M(QR), year]	64.5 (18.25)	71.5 (9.75)	−1.784	0.075
Gender (M/%)	45 (50)	13 (65)	1.477^a^	0.224
Hypertension (cases/%)	46 (51.1)	15 (75)	–	0.080^c^
Diabetes (cases/%)	13(14.4)	8(40)	6.919^a^	0.009
Hyperlipidemia (cases/%)	8 (8.9)	4 (20)	–	0.226^c^
Smoking history (cases/%)	18 (20)	8 (40)	3.626^a^	0.057
Drinking history (cases/%)	13 (14.4)	4 (20)	–	0.508^c^
TC [( $\bar{x}$ ± s)，mmol/L]	4.16 ± 0.99	3.73 ± 0.87	1.796^b^	0.075
TG [M(QR), mmol/L]	1.08(0.77)	1.03(0.65)	−0.302	0.762
HDL-C [M(QR), mmol/L]	1.30 (0.56)	1.17 (0.46)	−0.926	0.354
LDL-C [M(QR), mmol/L]	2.36 (1.32)	2.19 (1.04)	−1.554	0.12
sdLDL-C [M(QR), mmol/L]	0.52 (0.41)	0.50 (0.32)	−1.027	0.304
Hcy [M(QR), mmol/L]	12.15 (4.52)	13.25 (5.63)	−1.446	0.148
YKL-40 [M(QR)，ng/mL]	42.81 (19.58)	54.71 (12.91)	−3.193	0.001
MMP-9 [M(QR), ug/mL]	163.66 (59.86)	195.58 (18.05)	−2.999	0.003

^a^ is the *χ*^2^ value of the *χ*^2^ test, ^b^ is the *t*-value of the two independent samples *t*-test, ^c^ is the *p*-value of the Fisher exact test, and the remaining statistics are the *z*-values of the Mann–Whitney U test.

#### 3.3.2. Analysis of risk factors for posterior circulation positive remodelling

The statistically significant variables in Table 4 were used as independent variables, and whether or not the posterior circulation was positively remodelled was used as the dependent variable in a one-way binary logistic regression analysis (Model C), which showed that diabetes (OR = 0.253, 95% CI: 0.087–0.739, *p* = 0.012), YKL-40 (OR = 1.061, 95% CI: 1.018 ~ 1.106, *p* = 0.005), MMP-9 (OR = 1.024, 95% CI: 1.008–1.041, *p* = 0.003) and integrated indicator (OR = 3.711, 95% CI: 1.921 ~ 7.168, *p* < 0.01) were risk factors for positive remodelling of the posterior circulation. After correcting for a history of diabetes (model D), the results still showed that integrated indicator (OR = 3.763, 95% CI: 1.884 ~ 7.517, *p* < 0.01) were independent risk factors for positive remodelling of the posterior circulation (Table 5).

**Table 5:** Binary logistic regression analysis of risk factors for posterior circulation positive remodelling.

	Model C			Model D		
Projects	OR	95% CI	Value of *p*	OR	95% CI	Value of *p*
Diabetes	0.253	0.087 ~ 0.739	0.012	–	–	–
YKL-40	1.061	1.018 ~ 1.106	0.005	1.064	1.017 ~ 1.112	0.007
MMP-9	1.024	1.008 ~ 1.041	0.003	1.024	1.007 ~ 1.041	0.006
Integrated indicators	3.711	1.921 ~ 7.168	<0.01	3.763	1.884 ~ 7.517	<0.01

Model C is a one-way logistic regression analysis, and Model D is after correction for a history of diabetes.

### 3.4. Reproducibility analysis

Reproducibility analysis showed that both physicians measured an intraclass correlation coefficient of 0.914 (95% CI: 0.693–0.978, *p* < 0.001) for individual measurements of intracranial artery diameter and a mean intraclass correlation coefficient of 0.955 (95% CI: 0.818–0.989, *p* < 0.001), demonstrating good agreement between the two physicians’ measurements.

## 4. Discussion

In this study based on patients with CSVD, 3D-slicer software was applied to the 3D reconstruction of TOF MRA raw sequences to derive the mean arterial remodelling index. The results revealed that serum YKL-40 and MMP-9 levels were significantly higher in patients with positive arterial remodelling than in those non-positive remodelling. As YKL-40 may be an upstream signalling molecule of MMP-9 and may interact, we combined the two variables by factor analysis into one integrated indicator before including it in the logistic regression. Logistic regression analysis of the 110 patients revealed that the integrated indicator of serum YKL-40 and MMP-9 levels were independent risk factors for positive intracranial arterial remodelling. The results after correcting for risk factors showed that the integrated indicator of YKL-40 and MMP-9 were independent risk factors for intracranial arterial remodelling. Higher integrated indicator of serum YKL-40 and MMP-9 levels are independent risk factors for positive remodelling of the posterior circulation. In summary, we inferred that YKL-40 and MMP-9 are involved to some extent in arterial remodelling, and their levels may be of diagnostic value in the presence or absence of positive remodelling in intracranial arteries. Due to the high cost of imaging tests and the tedious process of comprehensive assessment of vascular conditions, it would be more economical and convenient to use circulating concentrations of YKL-40 and MMP-9 to predict and evaluate intracranial arterial remodelling.

The intracranial arterial wall consists of three membranes: inner, middle, and outer. The inner membrane is divided from the middle membrane by the inner elastic membrane, which contains more vascular smooth muscle cells and macrophages, and is the main component involved in arterial remodelling (Ballester-Servera et al., 2022). Positive remodelling is mainly influenced by two factors; haemodynamics and inflammatory reaction. Persistent high wall flow shear causes the endothelium to produce inflammatory mediators such as nitrogen monoxide and interleukin 6 (IL-6) under long-term chronic pressure (Wang D. et al., 2022), which causes endothelial cell damage and smooth muscle cell apoptosis through a cascade reaction, prompting outward expansion of the vessel wall, resulting in positive remodelling (Myasoedova et al., 2018). Increased macrophage activation, inflammatory cell infiltration, and smooth muscle cell phenotypic changes can occur during the pre-remodelling period (Cai et al., 2021), causing more atherosclerotic plaque formation and eventually leading to outward remodelling of the vessel wall (Di Ieva et al., 2013).

Current studies suggest that MMP-9 affects positive arterial remodelling primarily through high wall flow shear, stimulating macrophage activation and causing the release of large amounts of MMPs, which stimulate MMP-9 expression (Frösen et al., 2019). MMP-9 acts as a signalling molecule to increase the expression of the interleukin family (e.g., IL-6, IL-8, etc.) and macrophage chemotactic protein 1 in vascular endothelial cells (SHIH et al., 2021), thus exerting a positive remodelling effect. Arterial remodelling can be reversed by antagonising the MMP-9-related selective kinin B1 receptor and downregulating ERK/AKT phosphorylation, resulting in reduced MMP-9 expression (Rampa et al., 2021). High levels of circulating MMP-9 can cause intracranial remodelling of small arteries to occur first and connect with the remodelling of large and small arteries (Zhang et al., 2019).

YKL-40 is a circulating inflammatory factor detected in macrophages, smooth muscle cells, fibroblasts, and astrocytes (Yeo et al., 2019). Several studies have reported that YKL-40-mediated inflammatory remodelling is widely present in the cardiovascular and respiratory systems and may be a predictor of remodelling (Furukawa et al., 2019; Sun X. N. et al., 2022). In this study, YKL-40 levels were significantly higher in patients with positive intracranial arterial remodelling than in those without positive remodelling, possibly because YKL-40 acts as a pro-inflammatory signal to endothelial cells, regulates macrophage activation (Batinic et al., 2012), activates the protein kinase pathway, increases protease production, disrupts the internal elastic layer and collagen matrix, and causes positive intracranial arterial remodelling (He et al., 2013; Xu et al., 2019). In contrast, YKL-40 activates the MAPK/ERK pathway in macrophages, resulting in increased MMP-9 production, which is involved in smooth muscle cell migration in the mid-membrane and promotes atherosclerotic plaque formation (Iwata et al., 2009).

In this study, positive remodelling in the posterior circulation was associated with increased YKL-40 and MMP-9 levels, whereas no significant difference was observed in positive remodelling in the anterior circulation. This may be because positive remodelling is more likely to occur in the posterior circulation than in the anterior circulation (Wang S. et al., 2022), and elevated YKL-40 levels are more common in risk events, such as stroke and plaque in the posterior circulation (Chen et al., 2017). The simultaneous increase in the length and diameter of intracranial arteries, also known as intracranial arterial dolichoectasia, is an extreme manifestation of positive intracranial artery remodelling. Reportedly, posterior circulation intracranial arterial dolichoectasia is more common than anterior circulation intracranial arterial dolichoectasia, with basilar artery dilation accounting for 80% of all patients with intracranial arterial dilation (Del Brutto et al., 2017; Rathore et al., 2019). The pathogenesis of this is still unclear and may be because the basilar artery anatomy is more susceptible to haemodynamic influences and high-wall flow shear than other intracranial arteries, secretes more YKL-40 and MMP-9 and is, therefore, more prone to dilatory remodelling (Tanaka et al., 2013; Zhang et al., 2020).

This study has several limitations. Firstly, this study was a single-centre, cross-sectional study, and the conclusions were one-sided. Secondly, the sample size was small and prone to selective bias. Thirdly, the unit of YKL-40 is ng/mL, which is small; although the average value of the two experiments was taken, bias due to experimental conditions still cannot be excluded. Finally, no haemodynamically related indexes were included. In the future, a larger sample will be included in this study to follow up patients and explore the changes in serum YKL-40 and MMP-9 levels during different arterial periods to further clarify their role in the positive remodelling of intracranial arteries.

In conclusion, YKL-40 and MMP-9 may be involved in the development of positive intracranial arterial remodelling and may be serological markers of positive intracranial arterial remodelling, which could be used as targets for future research and may provide ideas for the prevention and treatment of patients with positive intracranial arterial remodelling.

## Data availability statement

The original contributions presented in the study are included in the article/supplementary material, further inquiries can be directed to the corresponding authors.

## Ethics statement

The studies involving human participants were reviewed and approved by the Ethics Committee of the Second Affiliated Hospital of Zhengzhou University. The patients/participants provided their written informed consent to participate in this study. Written informed consent was obtained from the individual(s) for the publication of any potentially identifiable images or data included in this article.

## Author contributions

MT contributed to study conceptualization and design. MT and DZ contributed to data collection, data processing and analysis, and article revision. MT, DZ, and JH drafted the manuscript. JH, HB, and QL critically revised the study. HB, QL, and HX supervised the study. HB and HX provided financial support. All authors contributed to the article and approved the submitted version.

## Funding

This study was supported by the Henan Province Medical Science and Technology Tackling Program Project (No. LHGJ20190314).

## Conflict of interest

The authors declare that the research was conducted in the absence of any commercial or financial relationships that could be construed as a potential conflict of interest.

## Publisher’s note

All claims expressed in this article are solely those of the authors and do not necessarily represent those of their affiliated organizations, or those of the publisher, the editors and the reviewers. Any product that may be evaluated in this article, or claim that may be made by its manufacturer, is not guaranteed or endorsed by the publisher.

## Abbreviations

CSVD: Cerebral small vessel disease
BAR: brain arterial remodelling
OR: odds ratio
CI: confidence interval
MRI: magnetic resonance imaging
MRA: magnetic resonance angiography
TC: total cholesterol
TG: triacylglycerol
HDL-C: high-density lipoprotein cholesterol
LDL-C: low-density lipoprotein cholesterol
sdLDL-C: small and low-density lipoprotein cholesterol
Hcy: homocysteine
TOF MRA: time-of-flight magnetic resonance angiography
MMPs: matrix metalloproteinases
3D: three-dimensional
